# *Clear, easy, plain*, and *simple* as keywords for text simplification

**DOI:** 10.3389/frai.2022.1042258

**Published:** 2022-11-30

**Authors:** Sara Vecchiato

**Affiliations:** Department of Languages and Literatures, Communication, Education and Society (DILL), University of Udine, Udine, Italy

**Keywords:** plain language, easy language, clear writing, text clarity, text simplification, discursive ergonomics, discourse and writing, text complexity

## Abstract

In this paper, we distinguish between four interconnected notions that recur in the literature on text simplification: *clarity, easiness, plainness*, and *simplicity*. While *plain language* and *easy language* have both been the subject of standardization efforts, there are few attempts to define *text clarity* and *text simplicity*. Indeed, in the definition of *plain language, clarity* has been favored at the expense of *simplicity* but is employed as a self-evident notion. Meanwhile, *text simplicity* suffers from a negative connotation and is more likely to be defined by its antonym, *text complexity*. In our analysis, we examine the current definitions of *plain language* and *easy language* and discuss common definitions of *text clarity* and *text complexity*. We propose a model of *text simplification* that can clarify the transition from specialized texts to plain language texts, and easy language texts. It is our contention that text simplification should be placed in a more general framework of *discursive ergonomics*.

## Introduction

The movement of opinion known as *plain language* has gained attention in many countries (Schriver, 2017; Clerc, 2022) and has popularized several notions for identifying what we may call reader-friendly writing. Among these labels, we find four recurring notions: *clarity, easiness, plainness*, and *simplicity*. These are semantically close notions that are sometimes employed interchangeably and may generate ambiguity, for example, Naderi et al. (2019) use the phrase *plain language* to refer to language used for people with disabilities and differentiate it from *easy language*, which they use for language for people with generic reading difficulties, contrary to Baumert (2016) and Maaß (2020). Moreover, even the current conceptualization of *text simplification* does not satisfy some specialists (Garbacea et al., 2021).

The goal of this article is to answer these research questions:

1. How are these notions related and how do they differ?
2. How can considering these four notions offer a better conceptualization of *text simplification*?

In fact, much effort has been devoted to developing a standard definition of *plain language* (Balmford, 2018); similar effort has been made with regard to proposing international standards for *easy language* (Lindholm and Vanhatalo, 2021). Conversely, there have been few attempts to define *text clarity*, with the consequence that some confusion persists regarding its meaning (Gracie, 2017). There have been even fewer attempts to define text *simplicity*, which seems rather defined by its antonym, *text complexity*. However, text complexity does not have an established definition either (Tolochko and Boomgaarden, 2019).

This paper is structured as follows: First we will discuss the definitions of *plain language, easy language, text clarity*, and *text complexity*; then, we will briefly present a modeling of the transition from *language for special purposes* to *plain language* and *easy language*. In this modeling, the tension between complex and simple text is articulated along different aspects of *text clarity*. Since *plain language* is an international movement, we will consider the English, French and German languages in our examples.

## *Plain language* as clear (and simple) language

In the 2000s, plain language specialists felt the need for a standard definition of this notion. In order to achieve this, the Plain Language Association InterNational formed a working group (James, 2009), which discussed several proposals. Cheek (2010) grouped the existing definitions into three categories. The first category consists of readability tests: These are methods based on objective indicators, such as the number of words per sentence; the second category consists of recommendations on wording to be chosen or avoided, such as word choice, sentence length, and content planning; the third category consists of definitions based on message outcome. The PLAIN working group favored the latter approach (Balmford, 2018). Its definition characterizes *plain language* as being sufficiently “clear” for a desired effect to occur:^1^

1) *Communication is in* plain *language if its wording, structure and design are so* clear *that the intended audience can easily find what they need, understand what they find, and use that information* (International Plain Language Federation, 2019).

In the French and German versions of this definition, the wording is slightly different. In German (2), the equivalent of *plain language* is *einfache Sprache* “simple language” (Baumert, 2016). Instead, the French label is *langage clair* “clear language” (Krieg-Planque, 2020). Since a literal translation would create a tautology, the second *clear* has been deleted (3):

2) *Eine Mitteilung ist in* einfacher *Sprache gehalten, wenn ihre Sprache, ihre Struktur und ihr Design so* klar sind, dass die gemeinten Leser^*^innen relevante Informationen leicht finden, verstehen und anwenden können.
  “Communication is considered to be in simple language, if its wording, its structure and its design are so clear that the intended readers can easily find, understand and use the relevant information” (International Plain Language Federation, 2019, translation is ours).
3) *Une communication est en langage* clair *si les mots et les phrases, la structure et la conception permettent au destinataire visé de facilement trouver, comprendre et utiliser l'information dont il a besoin*.
  “Communication is in clear language if its words and sentences, its structure and design allow the intended recipient to easily find, understand and use the information they need” (International Plain Language Federation, 2019, translation is ours).

Both *clarity* and *simplicity* are part of the dictionary meanings of *plain* (Merriam-Webster, 2022): In fact, they are present in the synonymic pair *clear and simple language*, which is attested as a paraphrase of *plain language* (Economic and Social Committee, 1995). However, there has been an evolution over time: according to Ngram Viewer (Michel et al., 2011), the use of this locution peaked in the 1940s and declined from the 1960s onward^2^. Schriver (2017, p. 345) recalls that initially *plain language* was indeed described as “simple and direct style,” but this characterization did not incorporate the innovations brought about by information design. Moreover, *simplicity* has suffered from a negative connotation and has met with opposition (Kimble, 1992b; Coleman, 1998): In fact, this notion is often mentioned in the literature, but one must continually take account of its negative counterpart:

4) *Plain language is clear language. It is* simple *and direct but not* simplistic *or patronizing* (Plainlanguage.gov, 2022).

We can infer that in defining the notion of *plainness, clarity* has displaced *simplicity*.

## *Easy language*: when *plain* is not enough

In the definitions (1–3), the parameter for quantifying *clarity* is *ease*. The adjective *easy* is opposed to *difficult* and refers to the absence of effort (Merriam-Webster, 2022). Indeed, cognitive psychology has long investigated the relationship between textual comprehension and cognitive effort: The perception of difficulty seems to be related to the relationship between effort and result (Kintsch, 1998, 2018). It is not surprising, then, that *ease* has become a category of its own: *easy language*.

The main characteristic of *easy language* is its target audience, which is people with disabilities. Among the best-known programs is that on *easy-to-read information* by Inclusion Europe (2009). Inclusion Europe proposes a definition of this type of writing, drafting it in an *easy-to-read* form. In this definition, a text is *easy* because it is visually *clear* and structurally *simple*:

5) Easy to read is information that is written in a simple way so that people with **intellectual disabilities** can understand it. It is important to use simple words and sentences. If there are words that are difficult to understand, an explanation is provided. The text needs to be clear to see (…) (Inclusion Europe, 2019).

Here, structural *simplicity* is used as a proxy for *easiness*, just as *complexity* is commonly used as a proxy for *difficulty*. For example, according to Hansen-Schirra et al. (2020, p. 18) *easy language* would fit into the extreme end of a difficulty spectrum, with the *specialized text* at the opposite end. Specialized text and *easy language* text are contrasted along two parameters: *complexity* and *comprehensibility*. The *plain language* text and the *common language* text lie between these two opposing poles, along an axis of progressive simplification.

While text simplification for the public has met with opposition, resistance toward simplified forms for people with disabilities goes as far as stigma: For this reason, Maaß (2020) argues for the creation of an intermediate category, *Easy Language Plus*.

## What exactly is *text clarity*?

Although it is often used as a self-evident notion, the conceptualization of *clarity* can be problematic (Swiggers, 1987; Meschonnic, 1997; Gracie, 2017). As to professional writing, some authors speak of *clear writing* (Gunning, 1983; Gottlieb, 1992; Kimble, 1992a; Ragins, 2012), others of *(text) clarity* (Michaelson, 1949; Walton, 1983; De Vries, 2002; Bischof and Eppler, 2011). We will treat these expressions as synonyms.

Among its meanings, the adjective *clear* is synonymous with *transparent, plain*, and *unmistakable* (Merriam-Webster, 2022). Moreover, Emig (1977, p. 126) associates *clarity* with the avoidance of ambiguity, which she considers a trigger for misunderstanding:

6) Clear writing by definition is writing which signals without ambiguity the nature of conceptual relationships, whether they be coordinate, subordinate, superordinate, causal, or something other.

Coleman (1998, p. 393) contrasts *clarity* with *precision*, attributing to the latter the function of avoiding ambiguity:

7) Clarity in fact includes a range of attributes: brief, simple, comprehensible and concise. Precision, on the other hand, refers to an exactness of expression, to an absence of ambiguity, an attempt to reduce contestability.

Beaudet (2001, p. 3) attempts a definition where *clarity* does not correspond to a set of attributes, but to the aim of the text itself (translation is ours):

8) the clarity of a text produced in the workplace corresponds to its effectiveness and is measured by the materialization, or not, of the action it was intended to provoke.

Among these three definitions, we opt for Beaudet (2001). In fact, Emig's and Coleman's definitions seem problematic to us for two different reasons: On the one hand, as we have argued elsewhere (Vecchiato, forthcoming), a text that is intended to be *clear* does not necessarily exclude the use of ambiguous expressions—such as a pun—because they can offer an accomplished synthesis of the text's content. Regarding Coleman's definition, on the other hand, we have misgivings about the necessarily “short” nature of a *clear* text: A text can be reasonably long if more words are needed to adequately explain a concept. Beaudet's definition seems more convincing to us, because the label *clear* is attributed to a text *ex post*: In other words, a text is “clear” if it has been understood.

Furthermore (*ibid*., p. 12), Beaudet emphasizes the dimension of the text's adequacy to the recipient—a dimension we approximate to *ergonomics* (translation is ours):

9) [C]larity is not a property of thought or language, *per se*, but the result of the match between the language strategies used and the communication situation.

Beaudet (2001) includes other related concepts in the scope of clarity, such as *readability, intelligibility*, and *coherence*. Recently, Labasse (2008) has taken up these concepts in the light of research in cognitive psychology. In his model, clarity or “intelligibility” takes different labels depending on the level of text information processing. At the *acquisitive* level, that is, the processing of vocabulary and syntax, we will speak of *readability*; at the *logical* level, of *coherence*; and at the *figurative* level, coinciding with the possibility of constructing a mental image of the text, of *representability*.

## *Text simplicity* and *text complexity*

As stated above, the notion of *simplicity* rather appears in the literature as an antonym of *complexity*. Therefore, if we start from this second notion, we must first distinguish the dimension of language from the dimension of text. In fact, the phrase *linguistic complexity* generally refers to a characteristic inherent in language as a system (Dahl, 2004). By contrast, *text complexity* concerns the speaker's use of the language (Saussurean *parole*, see Pallotti, 2015, p. 120).

As we have seen, *complexity* is used as a proxy for readers' *difficulty* in coping with it. In this regard, Miestamo (2008, p. 23) identifies two approaches to complexity: In the “absolute” approach, the complexity of language and text is addressed as an objective property, while in the “relative” approach, complexity is seen as cognitive cost/difficulty. In their definition of *text complexity*, Mesmer et al. (2012, p. 236) keep the two notions distinct, while Temnikova (2012, p. 34) treats them as synonyms, and Fisher and Frey (2014, p. 237) consider textual complexity as a set of features encompassing those of difficulty. According to Pallotti (2015, p. 118) it is a matter of polysemy: *Complexity* has three main meanings, and only meanings 2 and 3 are related to *difficulty*:

10) 1. *Structural complexity*, a formal property of texts and linguistic systems having to do with the number of their elements and their relational patterns; 2. *Cognitive complexity*, having to do with the processing costs associated with linguistic structures; 3. *Developmental complexity*, i.e., the order in which linguistic structures emerge and are mastered in second (and, possibly, first) language acquisition.

It is important to properly assess the complexity of a text, when deciding on its destination. In fact, we have suggested (Vecchiato and Gerolimich, 2013) the term *hypercomplexity* to describe choices, made from several linguistic options, which are “too difficult” for a specific text type. Structural complexity virtually covers all textual levels, that is, lexicon, syntax, cohesion, and discourse (Dascalu et al., 2018).

Among the best-known models for measuring text complexity is the US *Common Core State Standards in English Language Arts* (CCSSO and NGA, 2010). This model represents text complexity as a triangle, each side of which represents a set of factors: Quantitative factors, such as word length; Qualitative factors, such as levels of meaning; Reader and task considerations, such as prior knowledge. The Common Core has been criticized by some scholars because it devotes much more attention to the first two factors than to the last one (Mesmer et al., 2012; Fisher and Frey, 2014). Both Mesmer et al. (2012) and Fisher and Frey (2014) have proposed returning to the RAND Reading Study Group's heuristic for reading comprehension (Snow, 2002): RAND's heuristic is also tripartite, yet is based on the Text, the Reader, and the Reading activity. Mesmer et al. (2012, p. 236) clarify the connections between the three parts of this model in the figure to emphasize that “No element (…) occurs in isolation”. We shall expose presently a modeling of *text simplification* along a similar principle.

## Discussion: *Text simplification* and *discursive ergonomics*

Formal processes of *text simplification* are varied (Siddharthan, 2014; François, 2018; Garbacea et al., 2021; Ermakova et al., 2022). In this regard, Garbacea et al. (2021) emphasize the persistent ambiguity of this phrase, which can refer to different linguistic levels, that is, lexical, syntactic, and semantic, and the way it is conducted, whether manual or automatic, etc. While we will not deal here with the way text simplification is conducted, our goal is to provide a simplification model that considers all levels of structural complexity.

In order to contextualize text simplification, we suggest framing it within a more general framework of *discursive ergonomics* (Delavigne, 2019). *Ergonomics* is associated with document design, which allows for a “clarification of communicative expectations” (Romain et al., 2022, p. 113). In fact, as Py noted (Py, 1994), *simplification* is typical of *asymmetrical* communicative exchanges characterized by a pedagogical contract (“didacticité” as defined by Moirand, 1993). However, as we have seen, readers may reject simplification if they feel belittled. It is therefore necessary to calibrate choices according to the relationship one wants to establish with the reader. Therefore, linguistic matching or *tailoring* (Schillinger et al., 2021, p. 6) is a form of discursive ergonomics.

**Figure 1:**
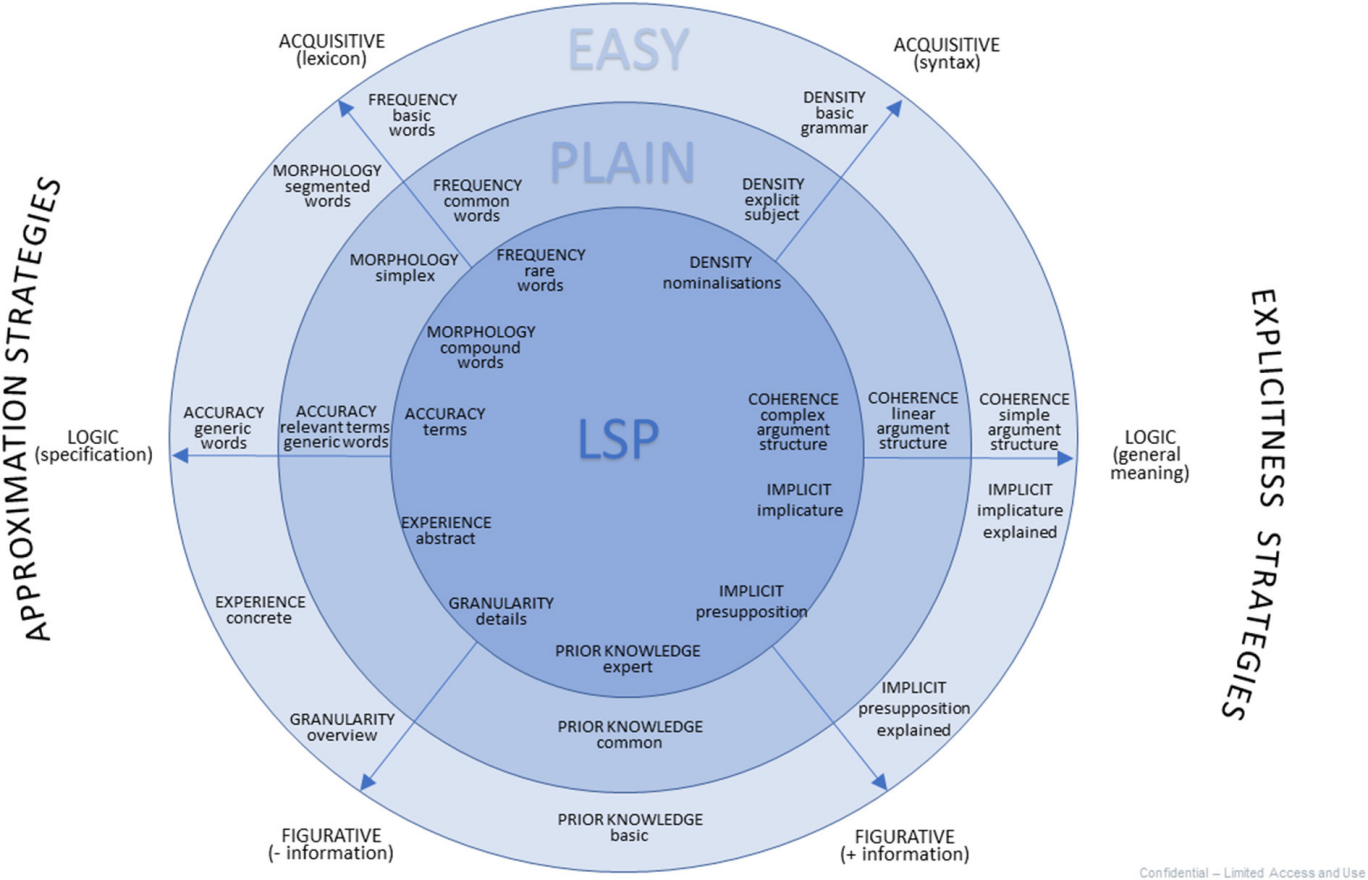
Text simplification wheel.

Our model highlights the links between levels of *structural complexity* and levels of *clarity*. Hence, we propose a diagram in Figure 1. In the center, we have the most complex text type, namely LSP (language for special purposes) texts. In an outer circle, we find plain language texts, and in a circle even further out, easy language texts. Simplification operations are split in two groups (Vecchiato, forthcoming): on the one hand, operations based on *approximation* (Bat-Zeev Shyldkrot et al., 2010), on the other, those based on *explicitness* (Sbisà, 2007). Both reduce the density of the text, but they do so over three levels of cognitive information processing (*acquisitive, logical*, and *figurative*).

In a study we conducted on popularization in the medical field (Vecchiato, forthcoming), we pointed out that in the transition from medical textbook to patient brochure, certain trends are constant. On the lexical level, long and compound words, and low-use “collateral terms” (Serianni, 2007, p. 82) give way to shorter, more frequent words, possibly segmented to allow the reader to easily identify morphemes. Some technical terms are retained, as they carry relevant content; in contrast, writing in easy language may further restrict their use. On the syntactic level, “condensed” sentences (Kocourek, 1982, p. 59) based on nominalization give way to less complex sentences. On the logical and figurative level, presuppositions, inferences, and more generally the communicative macro-act, are made explicit, often in the form of a question that the paragraph or text answers (Vecchiato et al., 2022). Overall, only relevant information (Wilson and Sperber, 2012) is selected.

## Conclusion

In this article, we pointed out that, in the definition of *plain language*, the notion of *simplicity* has been sidelined in favor of clarity, although simplicity recurs in research on *easy language*. Furthermore, we opted for the definition of *text clarity* proposed by Beaudet (2001) and integrated it with Labasse's (2008) model, which complexified the concept by identifying a different type of intelligibility (*readability, coherence, representability*) depending on the level of information processing (*acquisitive, logic, figurative*). Finally, we adopted Pallotti's (2015) distinction between structural complexity, cognitive complexity and developmental complexity. Therefore, we attempted a text simplification model that would integrate the different levels of intelligibility with various simplification steps. This “wheel” diagram should help writers to choose what level and what kind of simplification may work best. We emphasize the fact that text simplification should be conceptualized in a framework of *discursive ergonomics*.

## Data availability statement

The original contributions presented in the study are included in the article, further inquiries can be directed to the corresponding author.

## Author contributions

The author confirms being the sole contributor of this work and has approved it for publication.

## Funding

This study was funded by the Department of Languages, Literatures, Communication, Education and Society (DILL), University of Udine, Udine, Italy.

## Conflict of interest

The author declares that the research was conducted in the absence of any commercial or financial relationships that could be construed as a potential conflict of interest.

## Publisher's note

All claims expressed in this article are solely those of the authors and do not necessarily represent those of their affiliated organizations, or those of the publisher, the editors and the reviewers. Any product that may be evaluated in this article, or claim that may be made by its manufacturer, is not guaranteed or endorsed by the publisher.

## Footnotes

^1^In the examples, underlines are ours, while bold words are in the original; indents have been deleted for reasons of space.

^2^According to Ngram Viewer, the peak occurred in the 1940s for *langue claire et simple* and in the 2000s for *langage clair et simple*, while it occurred in the 1940s for *klare und einfache Sprache*.
